# Efficacy of Low-Dose Isotretinoin in the Treatment of Rosacea: A Systematic Review and Meta-Analysis

**DOI:** 10.7759/cureus.57085

**Published:** 2024-03-27

**Authors:** Ahmad Assiri, Alhassan H Hobani, Hanan A AlKaabi, Mohammed E Mojiri, Sarah A Daghriri, Osama A Suwaid, Mohammed I Alameer, Mohammed M Akkam, Mohammed A Alamir, Ali A Albarr, Mohammed R Alshaikh, Ali M Sumayli, Fatimah M Akkam, Hanin A Hakami

**Affiliations:** 1 Dermatology, Jazan University, Jazan , SAU; 2 College of Medicine, Jazan University, Jazan, SAU; 3 College of Medicine, King Khalid University, Abha, SAU; 4 Family and Community Medicine, Jazan University, Jazan, SAU

**Keywords:** low dose isotretinoin and rosacea, effectiveness of isotretinoin in treating rosacea, isotretinoin treatment of rosacea, isotretinoin and rosacea, low dose isotretinoin

## Abstract

Rosacea is a common cutaneous condition caused by persistent, recurring lesions in facial skin vessels. It is a chronic skin condition with a variety of clinical symptoms and an unknown cause. Rosacea begins with the widening of capillaries and a flushed appearance. Following that, telangiectasia appears, and reddened patches persist, particularly on the cheeks and nose. Erythema persists due to repeated vasodilation and telangiectasia. In addition, skin inflammation manifests as papules, pustules, lymphedema, and fibrosis. Despite recent advances in treatment, rosacea, a chronic inflammatory relapsing central facial dermatosis, can be extremely difficult to manage.

The purpose of this meta-analysis and systematic review was to evaluate the effectiveness of low-dose isotretinoin in the treatment of rosacea.

Following the guidelines set forth by the Preferred Reporting Items for Systematic Review and Meta-Analysis (PRISMA), the researcher employed the following search terms in the EMBASE, Web of Science, PubMed, Cochrane Library, and Google Scholar databases to provide a therapeutic update relevant to clinical practice: "low dose isotretinoin," "isotretinoin and rosacea," "isotretinoin treatment of rosacea," and "effectiveness of isotretinoin in treating rosacea". The search was carried out by the researcher for articles published from February 2019 to February 2024. The articles included were all published in the English language.

The overall frequency of patients with adverse events differed significantly between the groups treated with low-dose isotretinoin and the comparators (minocycline, pulsed dye laser, evening primrose oil, *Lactobacillus plantarum*, doxycycline, combined dose or placebo) (0.80, 95% CI 0.73 to 0.88, p = 0.0001). Sub-group analysis indicated that there was a difference between the interventions used in the treatments all in favor of low-dose isotretinoin treatment. The results showed that the moderate group had RR: 0.76, 95% CI: 0.44-1.30, I2 = 0%; the mild group had RR: 0.94, 95% CI: 0.56-1.57, I2 = 0%; and the group with severe rosacea had RR: 0.73, 95% CI: 0.47-1.13, I2 = 0%.

According to this study, rosacea can be treated effectively with low-dose isotretinoin even in patients at severe stages of the disease by using the recommended dose once a week. Further, the intervention has also been shown to have fewer side effects on the patients. Therefore, this study recommends randomized controlled trials to be done to fully investigate the best combination options for isotretinoin on mild to severe rosacea based on the fact that some of the treatments combined have shown to be effective on treatment.

## Introduction and background

Rosacea is a common cutaneous condition caused by persistent, recurring lesions in facial skin vessels [1]. It is a chronic skin condition with a variety of clinical symptoms and an unknown cause [2]. Rosacea begins with the widening of capillaries and a flushed appearance [3]; following that, telangiectasia appears, and reddened patches persist, particularly on the cheeks and nose. Erythema persists due to repeated vasodilation and telangiectasia [4]. Environmental factors that can exacerbate rosacea include heat, UV light, spicy food, alcohol consumption, and possibly smoking [5]. Although rosacea progresses differently in each person, papules, pustules, and rhinophyma are typical stages [6]. Rosacea affects women more frequently [7] due to the application of various cosmetics without observing their side effects [8]. Population-based data generally suggest a genetic predisposition for rosacea, and numerous extrinsic and intrinsic factors may be correlated with the disease's phenotypic expression [9]. According to estimates, between 1% and 10% of people have rosacea, and the condition is less common in people with darker skin tones [9]. It is estimated that 14 million Americans suffer from rosacea [10]. However, the frequency of rosacea in the Middle East is unknown [11]. Furthermore, the quality of life of people with rosacea is lower compared to that of the general population [12]. Even with recent advancements in treatment, rosacea, a chronic inflammatory relapsing central facial dermatosis, can still be extremely challenging to manage. Oral isotretinoin, also known as 13-cis-retinoic acid (13-CRA), is a vitamin A derivative that can gradually clear up moderate-to-severe acne [13]. The general consensus is that 13-CRA serves as a precursor to isomers of 9-CRA and/or all-trans-retinoic acid, which can bind to and activate the retinoid X and/or retinoic acid receptors [14]. However, isotretinoin may interfere with gene transcription and other cellular processes. Isotretinoin has the potential to reduce inflammation in a subset of patients by inhibiting the mammalian target of rapamycin complex 1 (MTORC1) [15].

However, isotretinoin is a highly successful rosacea treatment when taken as prescribed. It functions by shrinking sebaceous gland size, lowering sebum excretion, controlling cell division, and lowering keratinization. Because isotretinoin changes the follicles' microenvironment, it can reduce the amount of *Cutibacterium acnes* rosacea. Because of its antineoplastic qualities, it also has anti-inflammatory and immunomodulatory qualities, which make it a useful treatment for a variety of skin conditions [16]. These characteristics also include limiting the inflammatory cytokine response and decreasing monocyte toll-like receptor 2 expression [16]. Other research, such as the Australian study by Rademaker, revealed that isotretinoin is widely utilized for treating a variety of skin disorders such as inflammatory skin diseases, skin cancer, genodermatoses, and mild-to-moderate rosacea. It was observed in this particular study that using a lower dose of isotretinoin produced positive results with less severe side effects [17]. In light of this information, dermatologists should determine whether treating rosacea with a lower dosage of isotretinoin is effective. The aim of this research is to examine the most recent evidence-based data regarding the efficacy of treating rosacea with a lower dose of isotretinoin.

## Review

Literature search strategy

The Preferred Reporting Items for Systematic Reviews and Meta-analyses (PRISMA) guidelines were adhered to in this systematic review and meta-analysis. The researcher thoroughly searched through PubMed, EMBASE, Web of Science, Cochrane Library, and Google Scholar databases electronically for research articles published between February 2019 and February 2024 despite time constraints. The search strategy involved the use of search terms like "low dose isotretinoin," "isotretinoin and rosacea," "isotretinoin treatment of rosacea," and "effectiveness of isotretinoin in treating rosacea."

Inclusion and exclusion criteria

It included all relevant studies published between February 2019 and February 2024, randomized controlled trials (RCTs) with patients diagnosed with rosacea, studies that used different isotretinoin dosages or other treatments for rosacea treatment, and studies written in English. Excluded studies included duplicates, studies with a high potential for bias, and studies that didn't fit the requirements.

Selection of articles and data extraction

The primary search findings were imported into Mendeley to facilitate the removal of duplicate entries. The de-duplicated result was imported into Rayyan and independently screened using the study title and abstract. The complete text of each study was examined closely, and any discrepancies discovered during the screening phase were addressed by agreement among the authors.

Data were extracted using the primary author's name, publication year, country, dosage level, sample size, research design, and inclusion/exclusion criteria. Social factors such as age, social standing, and treatment method were considered during the data extraction process. For every study or trial that was included, two independent reviewers conducted the assessment of bias risk; discrepancies were settled by consensus and discussion.

Statistical analysis

The analysis utilized Resource Manager (Revman) 5.4.1 software (The Cochrane Collaboration). The heterogeneity of the study was evaluated using the Q-test and I2 statistic. The data from forest plots were shown with 95% confidence intervals (CI). The researcher calculated the odds ratios and standardized mean differences for the continuous and categorical outcomes of the funnel and forest plots using 95% confidence intervals. To assess the robustness of the results and the presence of publication bias, funnel plots and risk-of-bias analysis summary were used. Statistical significance was determined at p-value <0.05.

Results

**Figure 1:**
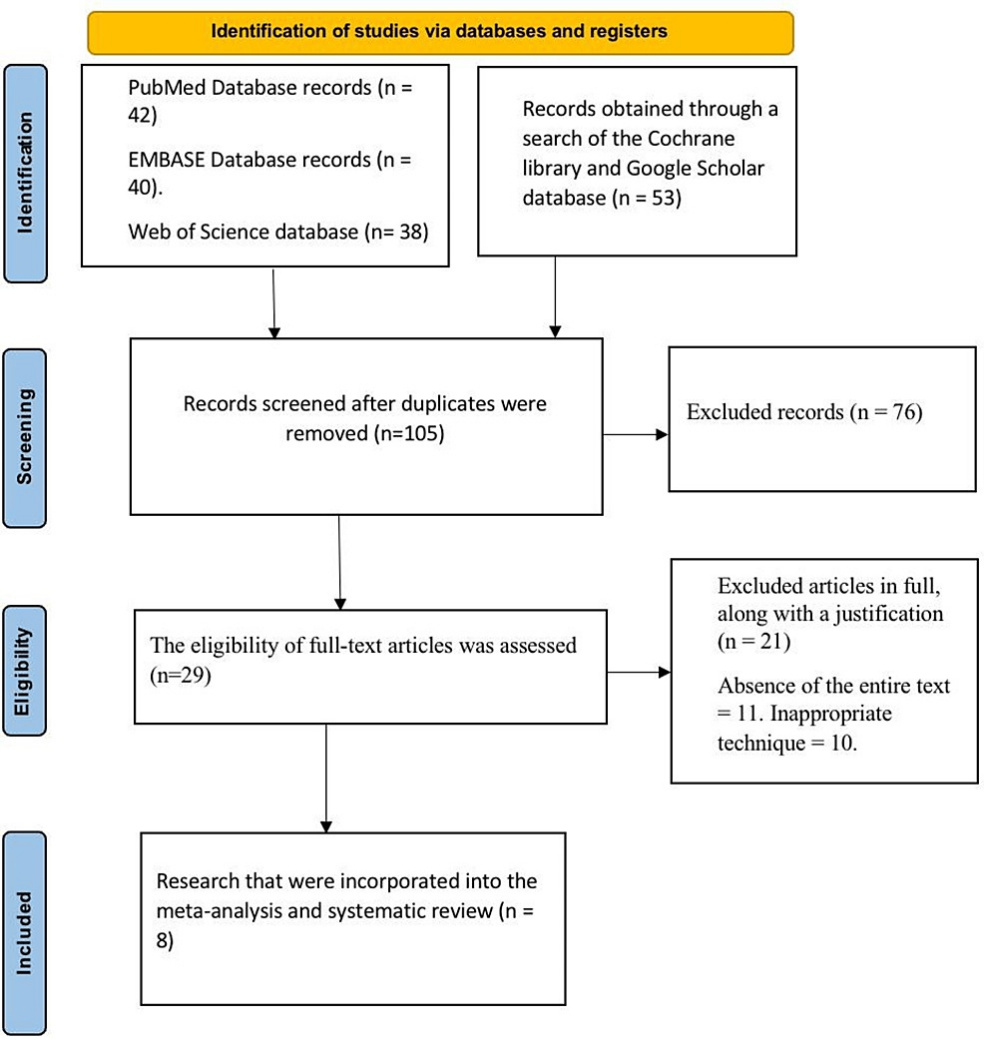
PRISMA flow diagram

Figure 1 shows a PRISMA diagram of the studies included in this analysis. A thorough search across several databases yielded 173 studies in total. Following the removal of duplicates, 105 were considered for further analysis. After 76 studies were excluded during the screening phase, 29 articles were selected for eligibility review. Twenty-one of them were excluded for failing to meet the predetermined requirements (inappropriate technique = 11, lack of full text = 10). Finally, eight studies were chosen for final inclusion.

Study characteristics

All eight studies were carried out as RCTs in the English language. Furthermore, the investigated studies were conducted in a variety of countries and regions. The findings show that the studies vary significantly in terms of year, location, sample size, design, dose, interventions, inclusions, and results. Table 1 displays these variations throughout each column. 

**Table 1:** Characteristics of the included studies

Author	Region	Sample size	Study design	Dose	Duration	Intervention	Inclusion	Outcome/Results
Shemer et al., 2021 [18]	Philippines	52	Retrospective randomized control trial	20 mg per week vs 100 mg per day	4 to 7 months	Low‐dose isotretinoin versus minocycline	Study design, dosage, intervention and diagnosis, and the duration of the assessment	A weekly dosage of 40 mg of isotretinoin is very effective in treating severe rosacea; improvements of more than 90% were seen in 62.5% of patients, while partial responses (improvements of 50% to 90%) were seen in 29.2% of patients. The efficacy of weekly low-dose isotretinoin (20 mg) and daily minocycline (100 mg) for mild-to-moderate rosacea appeared comparable. Furthermore, even in advanced cases of papulopustular rosacea, this study underscores the benefit of weekly low-dose isotretinoin treatment.
Ibrahim et al., 2021 [19]	Egypt	46	Randomized control trial	Isotretinoin (0.5 mg/kg/day) vs combined pulsed dye laser (0.25 mg/kg/day)	3 and 6 months	Pulsed dye laser and low-dose isotretinoin combined versus standard-dose isotretinoin	Study design, dosage, intervention and diagnosis, and the duration of the assessment	All parameters showed statistically significant improvements when isotretinoin and pulsed dye laser were combined at both evaluation times. Six patients (26%) in the isotretinoin group had flare-ups related to side effects, while no patients in the combined group did. The isotretinoin group had 13 patients (86%) who reported feeling parched, while the other group had five patients (21%). Notably, the ISO group received 100.4 ± 3.1 mg/kg of isotretinoin, while the isotretinoin and pulsed dye laser groups received significantly less (48.7 ± 5.7 mg/kg) (P
Yanfei et al., 2023 [20]	Xi'an, China	48	Retrospective randomized control trial	20 mg versus 10 mg per day	2-6 months	Isotretinoin	Study design, dosage, intervention and diagnosis, and the duration of the assessment	Between the two groups, there was no significant difference in the endpoint standard deviation values (2.21 ± 0.24 vs. 2.35 ± 0.46, p = 0.18) (p = 0.78), and the patients accepted both treatment plans. The most frequent adverse effect was cheilitis, but neither group reported any serious adverse events. Low doses of isotretinoin are effective for treating moderate to severe rosacea.
Kaźmierska et al., 2022 [21]	Poland	50	A randomized double-blind trial	0.5 mg isotretinoin/kg bw/day vs 2 capsules (2 × 510 mg)/day	9 months	Isotretinoin vs combined isotretinoin and evening primrose oil	Study design, dosage, intervention and diagnosis, and the duration of the assessment	Following the intervention, TEWL (p = 0.004), sebum (p < 0.001), and CORN (p = 0.015) exhibited significant decreases in the isotretinoin-treated group. A mix of Isotretinoin and evening primrose oil treatment resulted in a significant (p < 0.05) decrease in sebum and TEWL levels, but an increase (p = 0.017) in CORN levels from 42.0 ± 9.70 to 50.9 ± 10.4. Evening primrose oil supplements improved skin hydration while on isotretinoin therapy.
Liang et al., 2023 [22]	Shanxi China	105	Clinical randomized control trial	2 g/day vs 50 mg/kg/day	12 weeks	*Lactobacillus plantarum* vs isotretinoin	Study design, dosage, intervention and diagnosis, and the duration of the assessment	Using *L. plantarum* MH-301 in conjunction with isotretinoin may be the best way to treat acne vulgaris. The study indicates that *L. plantarum* can be used as an adjuvant to treat acne vulgaris.
Andrade et al. 2020 [23]	Brazil	39	Randomized control trial	0.3-0.4 mg/kg vs 100 mg/day	16 weeks	Isotretinoin vs doxycycline	Study design, dosage, intervention and diagnosis, and the duration of the assessment	For patients with rosacea, doxycycline improved ocular symptoms, meibomian gland dysfunction, and the ocular surface. Low-dose isotretinoin therapy did not cause any significant side effects in any of the subjects, although some patients experienced worsening symptoms and meibomian gland dysfunction when using doxycycline treatment.
Husein et al. 2021 [24]	Multiple countries	365	Randomized control trial; systematic review and meta-analysis	100 mg or 200 mg vs 0.3 mg/kg	16 weeks	Antimicrobial-dose doxycycline vs isotretinoin	Study design, dosage, intervention and diagnosis, and the duration of the assessment	Subgroup analysis of the studies comparing the active drugs (minocycline and isotretinoin; RR: 0.52, 95% CI: 0.17–1.63, I2 = 90%) revealed no distinction between the approaches.
Nooruldeen and Saeed 2021 [25]	Iran	40	Prospective clinical trial	0.5 mg/kg per day, split into two doses.	3 months	Placebo vs isotretinoin	Study design, dosage, intervention and diagnosis, and the duration of the assessment	Comparing the outcomes of the first, second, and third months of treatment showed a significant increase (p = 0.003) in the proportion of patients who had a full response by the end of the three months. Thirteen of the 15 patients who experienced a complete response were disease-free at the three-month follow-up, with only two of them experiencing a relapse. During the course of the treatment, all of the side effects of isotretinoin were mild and easily remedied; no serious side effects were reported. Treating rosacea with oral isotretinoin is both safe and effective.

Risk-of-bias assessment

**Figure 2:**
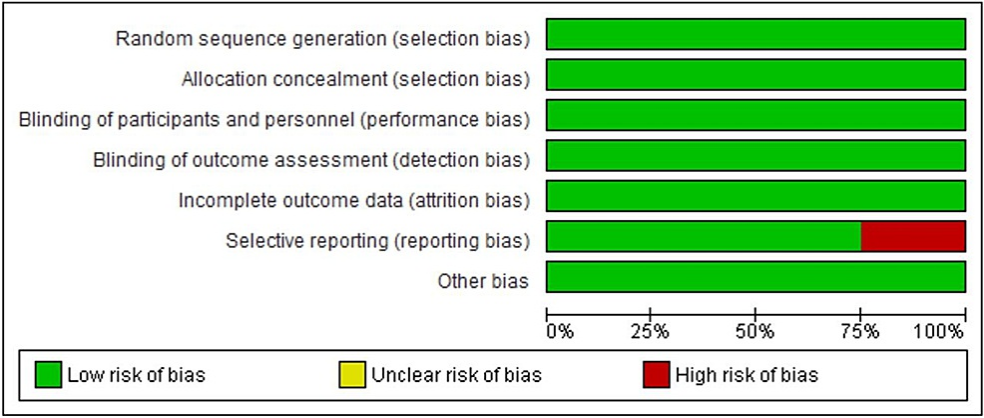
Summary of studies’ risk of bias of each item. A risk-of-bias summary is provided based on an examination of the risk-of-bias items and the researcher's evaluations.

Figure 2 shows information about the bias risk of the eight RCTs. The results show that the designs satisfied the requirements because there was little chance of bias in most of the studies. To mitigate bias related to selection, attrition, performance, and detection, a random sequence generator was utilized. However, only a minority of studies (25%) exhibited a high risk.

Meta-analysis of the efficacy outcome

**Figure 3:**
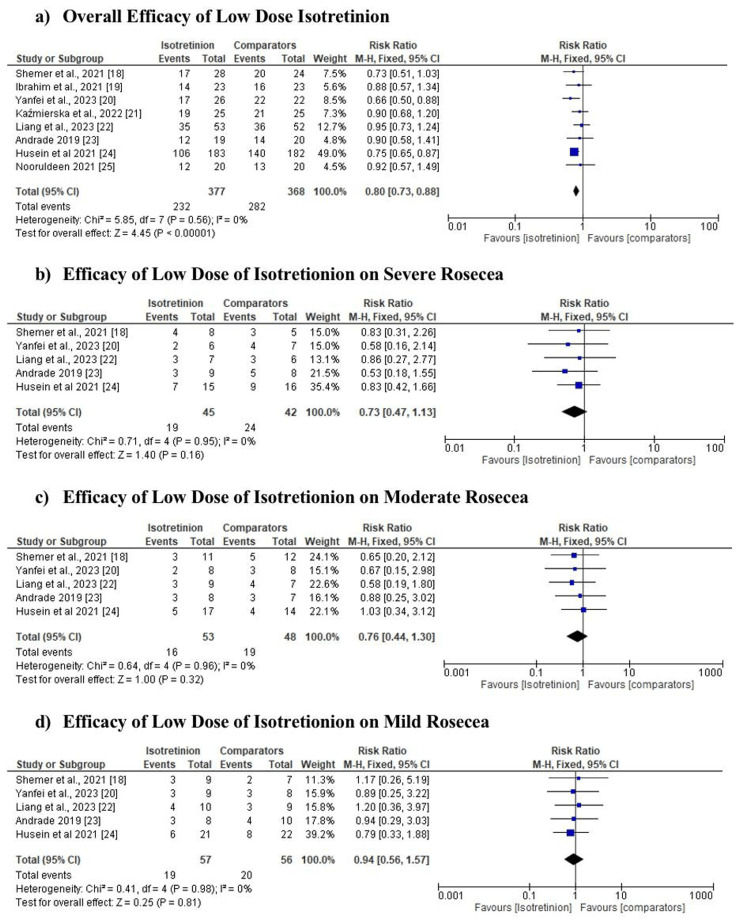
Efficacy of low-dose isotretinoin Source: Shemer et al. 2021 [18], Ibrahim et al. 2021 [19], Yanfei et al. 2023 [20], Kaźmierska et al. 2022 [21], Liang et al. 2023 [22], Andrade et al. 2020 [23], Husein et al. 2021 [24], Nooruldeen and Saeed [25].

Figure 3 shows group and sub-group analysis of low-dose isotretinoin efficacy in the treatment of rosacea. The overall frequency of patients with adverse events differed significantly between the groups treated with low-dose isotretinoin and the comparators (minocycline, pulsed dye laser, evening primrose oil, *Lactobacillus plantarum*, doxycycline, combined dose or placebo) (RR 0.80, 95% CI 0.73 to 0.88, p = 0.0001; Figure 3A). Sub-group analysis involved five studies that evaluated treatment outcome based on the level of rosacea treated (severe, moderate, and mild) [18,20,22-24]. Among the three sub-groups analyzed, there was a difference between the interventions used in the treatments all in favor of low-dose isotretinoin treatment. As per Figure 3C, the moderate group exhibited RR: 0.76, 95% CI: 0.44-1.30, I2 = 0%; the mild group displayed RR: 0.94, 95% CI: 0.56-1.57, I2 = 0% (see Figure 3D); and the severe group demonstrated RR: 0.73, 95% CI: 0.47-1.13, I2 = 0% (see Figure 3B).

Funnel plots

**Figure 4:**
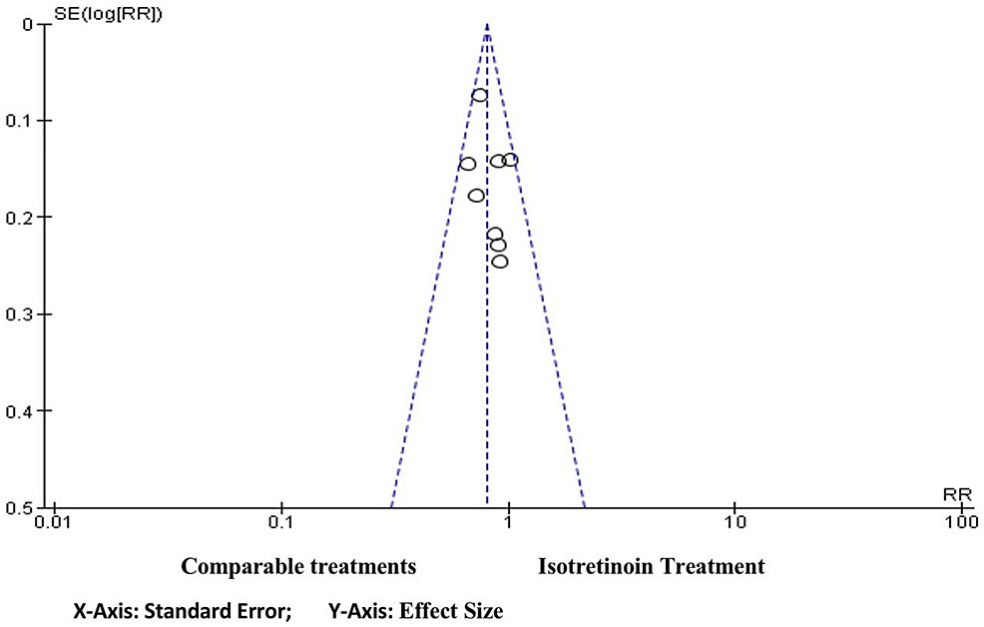
Funnel plot depicting publication bias. RR, relative risk; SE, standard error.

Figure 4 shows a funnel plot that illustrates the asymmetric distribution of effect sizes as a function of study precision with both sides having an unequal number of studies. The asymmetrical distribution of the sampled studies indicates that there is a probability of having a high risk of publication bias toward the right side which has more studies (five studies) compared to the left side which has only three studies.

Discussion

This systematic review and meta-analysis aimed to evaluate the effectiveness of low-dose isotretinoin in treating rosacea. The overall frequency of patients with adverse events differed significantly between the groups treated with low-dose isotretinoin and the comparators (minocycline, pulsed dye laser, evening primrose oil, *L. plantarum*, doxycycline, combined dose, or placebo) (0.80, 95% CI 0.73 to 0.88, p = 0.0001; Figure 3A) with outcomes favoring the group treated with low-dose isotretinoin. In consistency with findings obtained in this study, a study by Shemer and his friends found that isotretinoin at a dose of 40 mg weekly is very effective for severe rosacea. According to the study, 62.5% of patients experienced a complete response (improvement of over 90%), and an additional 29.2% experienced a partial response (improvement of 50% to 90%). The efficacy of 100 milligrams of minocycline per day and 20 milligrams of isotretinoin per week for mild-to-moderate rosacea was comparable. The research discovered that weekly administration of low-dose isotretinoin effectively treats papulopustular rosacea, even among individuals with advanced stages of the condition [18].

The findings from the sub-group analysis indicated that the interventions used in the treatments differed with almost all interventions in favor of low-dose isotretinoin treatment. However, in a study by Ibrahim et al., it was found that a combination of a proportion of isotretinoin and pulsed dye laser had a statistically significant positive change in all parameters (efficacy and safety) compared to isotretinoin used alone. In terms of adverse events, six patients (or 26%) in the isotretinoin group experienced flare, compared to none in the combined group. Further, in the isotretinoin group, 20 patients (86%) reported dryness, compared to five patients (21%) from the other group. Compared to the isotretinoin group (100.4 ± 3.1 mg/kg), the group receiving isotretinoin and pulsed dye laser combined received a lower cumulative dosage (48.7 ± 5.7 mg/kg) (p < 0.05) [19]. Furthermore, a study by Yanfei et al. unveiled that among the various doses of isotretinoin assessed for effectiveness, there were no notable differences between the two groups' endpoint standard deviation values (2.21 ± 0.24 vs. 2.35 ± 0.46, p = 0.18); hence, the patients approved both treatment plans (p = 0.78). However, the study noted cheilitis as the only most common side effect caused by higher quantities of isotretinoin, but neither group reported any serious adverse events. In summation, the study noted that low doses of isotretinoin are effective for treating moderate to severe rosacea [20].

Further, the findings from this study revealed a variation in the improvement of the patients diagnosed with severe rosacea based on the treatments applied to them (RR: 0.73, 95% CI: 0.47-1.13, I2 = 0%) with outcomes from the five studies [18,20,22,23,24] favoring the group treated with a low dose of isotretinoin. In alignment with these findings, a study conducted by Kaźmierska in Poland compared the effects of isotretinoin alone versus a combination of isotretinoin and primrose oil treatment. Significant reductions were noted in the isotretinoin-treated group: TEWL (p = 0.004), CORN (p = 0.015), and sebum levels (p < 0.001). However, in the combined treatment group of TEWL, isotretinoin, and evening primrose oil, sebum levels and TEWL notably reduced (p < 0.05) but CORN levels increased (p = 0.017) from 42.0 ± 9.70 to 50.9 ± 10.4 [21]. Conversely, Liang et al.'s research indicates that *L. plantarum* can be used as an adjuvant in the management of acne vulgaris. However, the best combination for treating acne vulgaris was found to be isotretinoin and *L. plantarum* MH-301 [22].

Nevertheless, the study found that in the treatment of both moderate and mild rosacea, low-dose isotretinoin was the most effective compared to other interventions (minocycline, pulsed dye laser, evening primrose oil, *L. plantarum*, doxycycline, combined dose, or placebo). This was shown by RR: 0.76, 95% CI: 0.44-1.30, I2 = 0% for moderate and RR: 0.94, 95% CI: 0.56-1.57, I2 = 0% for mild group, respectively. In support of these findings, a study by Andrade et al. found that among the patients treated for rosacea, none of them had any major side effects from low-dose isotretinoin therapy with patients exacerbating meibomian gland dysfunction and symptoms while using doxycycline treatment [23]. However, in a study by Husein‐ElAhmed and Steinhoff, no statistically significant relationship was found between the isotretinoin and minocycline interventions (RR: 0.52, 95% CI: 0.17-1.63, I2 = 90%) but the quantity of the isotretinoin used was not put into consideration [24]. In a separate study conducted by Nooruldeen and Saeed, the comparison of outcomes after the first three months of isotretinoin treatment revealed an increase in the proportion of patients achieving a complete response by the end of the three months (p = 0.003). However, there were only two of the 15 patients who experienced a complete response with a recurrence at the three-month follow-up, with the remaining 13 patients remaining disease-free. During the course of the treatment, all of the side effects of isotretinoin were mild and easily remedied with no serious side effects being reported. Common side effects of low-dose isotretinoin include dry lips, eyes, and skin. It can also cause mild irritation, increasing sensitivity to the sun, and an initial worsening of acne. Minocycline, on the other hand, frequently causes gastrointestinal symptoms like nausea and diarrhea, skin discoloration, vaginal yeast infections, and dizziness as adverse effects which are more severe considered to that of low-dose isotretinoin. Women with rosacea and who are of childbearing age are recommended to work closely with their healthcare provider to create a treatment plan that is safe and appropriate for their individual requirements, taking into account possible interactions between medications and pregnancy, as well as how hormone fluctuations may affect rosacea symptoms. Their research backs the findings revealed in this study in that treating rosacea with oral isotretinoin is both safe and effective [25]. Therefore, based on these findings, the study suggests that a low dose of isotretinoin administered on a weekly basis may be helpful for this condition at various severity levels.

## Conclusions

In summation, the study has shown that rosacea can be treated effectively with low-dose isotretinoin even in patients with advanced levels of the disease by applying the prescribed dose once a week. Further, the intervention has also been shown to have fewer side effects on the patients. Therefore, this study recommends RCTs be conducted to fully investigate the best combination options of the intervention for mild to severe rosacea based on the fact that some of the treatments combined have shown to be effective in the treatment of rosacea almost similar to that of low-dose isotretinoin.
